# Rifabutin Resistance Associated with Double Mutations in *rpoB* Gene in *Mycobacterium tuberculosis* Isolates

**DOI:** 10.3389/fmicb.2017.01768

**Published:** 2017-09-14

**Authors:** Wei Jing, Yu Pang, Zhaojing Zong, Jing Wang, Ru Guo, Fengmin Huo, Guanglu Jiang, Yifeng Ma, Hairong Huang, Naihui Chu

**Affiliations:** ^1^Department of Tuberculosis, Beijing Tuberculosis and Thoracic Tumor Research Institute, Beijing Chest Hospital Affiliated to Capital Medical University Beijing, China; ^2^National Clinical Laboratory on Tuberculosis, Beijing Tuberculosis and Thoracic Tumor Research Institute, Beijing Chest Hospital Affiliated to Capital Medical University Beijing, China

**Keywords:** tuberculosis, rifabutin, *rpoB*, double mutations, rifampicin

## Abstract

The objective of this study was to investigate the cross-resistance between rifampin (RIF) and rifabutin (RFB) among clinical *Mycobacterium tuberculosis* (MTB) isolates, and the correlations between specific *rpoB* mutations and the minimum inhibitory concentrations (MICs) of RIF and RFB. A total of 256 RIF-resistant isolates were included from the National Tuberculosis Clinical Laboratory in China. The MICs of MTB isolates against RIF and RFB were determined by using a microplate alamarBlue assay. In addition, all the MTB isolates were sequenced for mutations in *rpoB* gene. 204 out of 256 isolates (79.7%) were resistant to RFB, whereas 52 (20.3%) were susceptible to RFB. RIF-resistant/INH-susceptible (RR) group had a significant lower proportion of RFB-resistance than MDR- (*P* = 0.04) and XDR-TB group (*P* < 0.01). DNA sequencing revealed that there were 218 isolates (85.2%) with a single mutation, 26 (10.1%) with double mutations, and 12 (4.7%) without mutation in *rpoB* gene. Notably, although the single substitution of Leu511Pro, Asp516Gly, and His526Asn did not result in RFB resistance, 77.8% (7/9) of the MTB isolates with these double mutations became resistant to RFB. Compared with RR group (38.9%, 7/18), MDR-TB (63.5%, 106/167) had significantly higher proportion of isolates with mutations in codon 531 of *rpoB* gene (*P* = 0.04). Our data demonstrate that various *rpoB* mutations are associated with differential resistance to RIF and RFB. A single specific mutation in codons 511, 516, 526, and 533 was linked to the susceptibility to RFB for MTB, while the strains with these double mutations irrelevantly conferring RFB resistance produced RFB-resistant phenotype.

## Introduction

Tuberculosis (TB), caused by *Mycobacterium tuberculosis* complex (MTBC), is a major threat to public health worldwide (World Health Organization, 2016). In 2015, an estimated 10.4 million new TB cases emerged, and 1.4 million died of the disease globally (World Health Organization, 2016). Although the incidence and mortality rates of TB are slowing declining, the current advances in TB control remains impeded by the alarming increase in reports of drug-resistant TB, especially multidrug-resistant TB (MDR-TB), defined as resistant to at least rifampicin (RIF) and isoniazid (INH) (Gandhi et al., 2010; Jamieson et al., 2014). RIF is a cornerstone of TB treatment and is a key factor in determining the treatment efficacy of the treatment regimens (Somoskovi et al., 2001; Pang et al., 2013a). As more than 90% of RIF-resistant TB strains are simultaneously resistant to INH, RIF resistance is considered as a promising marker for the diagnosis of MDR-TB, and RIF-resistant cases are also eligible for MDR-TB treatment if the INH susceptibility is inaccessible in clinical practice (Garcia de Viedma et al., 2002; Pang et al., 2013a).

Rifabutin (RFB) is a semisynthetic derivate of rifamycin S (Marsili et al., 1981; Uzun et al., 2002), and is part of the rifamycin family together with RIF. Despite sharing several of the common properties of RIF, RFB exhibits more potent efficacy against *M. tuberculosis*, as well as *M. avium* complex and *M. leprae* (Kunin, 1996). In addition, due to fewer interactions with protease inhibitor drugs, RFB is recommended to be used as an alternative to treat MTB in HIV-infected patients (Berrada et al., 2016). Resistance to both RIF and RFB is largely associated with mutations in an 81-bp RIF resistance determining region (RRDR) between *rpoB* codons 507 and 533 of MTB (Rukasha et al., 2016), and mutations within codons 516, 526, and 531 are responsible for up to 90% of RIF-resistant strains (Pang et al., 2013a). High-level cross-resistance between the two rifamycins is noted by numerous studies (Beckler et al., 2008; Berrada et al., 2016; Rukasha et al., 2016), whereas not all mutations within the RRDR display the same loss of rifamycin susceptibility, and only some specific *rpoB* mutations confer RFB resistance in MTB (ElMaraachli et al., 2015). Hence, a detailed analysis of the association between RFB susceptibility and genetic mutations within *rpoB* gene would provide new insights for guiding RFB-based therapeutic regimens. In China, due to the limited data regarding this issue, RFB is not routinely used in the treatment of RIF-resistant TB cases, which make the cases lose the opportunity to access the potentially effective RFB. In this study, we aimed to investigate the cross-resistance between RIF and RFB among clinical MTB isolates. In addition, the correlations between specific *rpoB* mutations and the minimum inhibitory concentrations (MICs) of RIF and RFB were analyzed, which will lay a foundation to establish criteria to predict *in vitro* susceptibility of MTB against RFB by molecular detection of *rpoB* gene.

## Materials and methods

### Bacterial isolates

A total of 256 RIF-resistant isolates were included from the National Tuberculosis Clinical Laboratory, Beijing Chest Hospital in China. These strains were isolated from sputum samples collected from pulmonary TB patients between February 2015 and August 2015. The primary susceptibility to RIF was determined by conventional absolute concentration method on Löwenstein-Jensen (L-J) medium containing the corresponding anti-TB drugs according to the guideline of World Health Organization (WHO) (Zhang L. et al., 2014). The concentration of anti-TB drugs were as follows: RIF, 40 mg/L; INH, 0.2 mg/ml; streptomycin (SM), 10 mg/L; ethambutol (EMB), 2 mg/L; kanamycin (KAN), 30 mg/L; capreomycin (CPM), 40 mg/L; amikacin (AMK), 30 mg/L; ofloxacin (OFLX), 2 mg/L; levofloxacin (LFX), 2 mg/L. Multidrug-resistant TB (MDR-TB) was defined as the strains with resistance to both RIF and INH; extensively drug-resistant TB was defined as MDR-TB with additional resistance to any fluoroquinolone and to at least one of three injectable anti-TB drugs (ie, kanamycin, capreomycin, or amikacin). *All work with MTB was conducted in a biosafety level 2(BSL-2) laboratory under negative pressure system at Beijing Chest Hospital, which certified by Health Bureau of Beijing*. Ethics approval for this study was obtained from the Ethics Committee of Beijing Chest Hospital, Capital Medical University. Written informed consent was obtained from each participant.

### Determination of minimal inhibitory concentration (MIC)

The minimal inhibitory concentrations of RIF-resistant MTB isolates against RIF and RFB were determined by using a microplate alamarBlue assay as previously described (Zhang Z. et al., 2014). Briefly, the 4-week-old cultures were harvested from the surface of L-J medium, and the turbidity of cultures was adjusted to 1.0 McFarland standard. Prior to inoculation, the 1.0 McFarland cell suspension was diluted to 1:20 in Middlebrook 7H9 broth supplemented with 10% OADC. 100 μL of this inoculum was pipetted into the wells of the 96-well plate. After 7-day incubation at 37°C, 70 μL of AlamarBlue solution was added to each well, incubated for 24 h at 37°C, and assessed for color development. The bacterial growth was declared if the presence of the color change from blue to pink. MIC was defined as the lowest concentration of antibiotic that prevented the bacterial growth. The concentration gradient for each drug ranged from 0.0625 to 32 mg/L. *M. tuberculosis* H37Rv (ATCC 27249) was tested in all runs as a quality control. All experiments were performed in duplicate to access reproducibility. The critical concentration for RIF and RFB were 1.0 and 0.5 mg/L, respectively, as recommended by previous reports (Schön et al., 2013; Berrada et al., 2016).

### DNA sequencing

All isolates were subcultured on L-J medium for four weeks. Genomic DNA was extracted with the rapid boiling method. A 688 bp fragment of the *rpoB* gene containing the entire RRDR was amplified from the crude DNA prepared above (Caoili et al., 2006). The PCR mixture was prepared in a volume of 50 μL as follows: 25 μL 2 × PCR Mixture (CWBio, Beijing, China), 5 μL of DNA template and 0.2 μM of each primer set. The PCR amplification protocol consisted of a 5 min denaturation at 94°C, followed by 30 cycles of 1 min at 94°C, 1 min at 62°C and 1 min at 72°C and a final extension step at 72°C for 10 min. The purified amplification product was sent to Ruibio Company (Beijing, China) for DNA sequencing service. Mutations in *rpoB* gene were determined by alignment to the homologous sequences of the reference *M. tuberculosis* H37Rv strains using multiple sequence alignments (http://www.ncbi.nlm.nih.gov/BLAST).

### Statistical analysis

A chi-square test was used to determine whether there was significant difference in the proportions of RFB resistance among different MTB groups. In addition, paired comparisons in the correlations between resistant levels and mutant types were analyzed with paired chi-square test or Fisher's exact test. Statistical analysis was performed in SPSS 17.0 (SPSS Inc., USA). The difference was declared as significant if *P* value was lower than 0.05 for chi-square test, while for the paired comparisons, the difference was considered as significant if *P*-value was low than false discovery rate (FDR) for to reduce the false positive results. The FDR was calculated as previously reported (Benjamini and Hochberg, 1995).

## Results

### Cross-resistance between RIF and RFB

**Figure 1:**
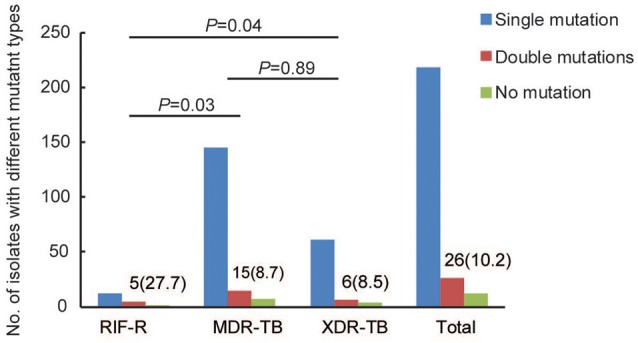
Distribution of MTB isolates with different mutation types among RR-, MDR- and XDR-TB.

A total of 256 RIF-resistant isolates determined by conventional method were enrolled in this study, including 18 RIF-resistant/INH-susceptible (RR) isolates (7.0%), 167 MDR-TB isolates (65.2%), and 71 XDR-TB isolates (27.7%) (Table S1). Out of these isolates, 204 isolates (79.7%) were resistant to RFB, whereas 52 (20.3%) were susceptible to RFB. We further analyzed the proportion of RFB-resistant MTB isolates among different drug susceptibility profile group. As shown in Table 1, the rates of RFB-resistance were 55.5% (10/18), 72.5% (121/167), and 87.3% (62/71) for RR-, MDR-, and XDR-TB groups, respectively. Statistical analysis revealed that RR group had a significant lower proportion of RFB-resistance than MDR- (*P* = 0.04) and XDR-TB group (*P* < 0.01). Similarly, there was significant difference between MDR- and XDR-TB group (*P* = 0.01) (Figure 1).

**Table 1:** Distribution of *rpoB* mutation and MICs of RIF and RFB.

**Codon**	**Nucleotide substitution**	**Amino acid change**	**No. of isolates**	**No. of RFB-resistant isolates (%)**	**MIC (mg/L)^a^**		**No. of isolates with different MICs**	**References^b^**
					**RIF**	**RFB**		
**SINGLE MUTATION**								
511	CTG-CCG	Leu-Pro	2	0 (0.0)	2	≤0.25	2	Kapur et al., 1994
513	CAA-AAC	Gln-Asn	1	1 (100.0)	≥32	2	1	Bahrmand et al., 2009
514	Ins TTC	Ins Phe	2	1 (50.0)	≥32	4	1	Kapur et al., 1994
					16	≤0.25	1	
516	GAC-GTC	Asp-Val	5	4 (80.0)	≥32	16	1	Kapur et al., 1994
					≥32	8	1	
					≥32	4	1	
					≥32	2	1	
					2	≤0.25	1	
516	GAC-TAC	Asp-Tyr	1	0 (0.0)	2	≤0.25	1	Kapur et al., 1994
	GAC-GGC	Asp-Gly	2	0 (0.0)	16	≤0.25	1	Hillemann et al., 2005
					2	≤0.25	1	
522	TCG-CAG	Ser-Gln	1	1 (100)	≥32	2	1	Yuen et al., 1999
	TCG-TTG	Ser-Leu	1	0 (0.0)	2	≤0.25	1	Bodmer et al., 1995
526	CAC-GAC	His-Asp	11	11 (100.0)	≥32	1	11	Kapur et al., 1994
	CAC-TGC	His-Cys	7	7 (100.0)	≥32	2	7	Kim et al., 1997
	CAC-TAC	His-Tyr	2	0 (0.0)	≥32	≤0.25	1	Kapur et al., 1994
					8	≤0.25	1	
	CAC-CGC	His-Arg	9	9 (100)	≥32	1	9	Kim et al., 1997
	CAC-CTC	His-Leu	6	0 (0.0)	≥32	≤0.25	1	Kapur et al., 1994
					16	0.5	1	
					8	≤0.25	1	
					4	≤0.25	1	
					2	≤0.25	2	
	CAC-GAC	His-Asp	1	0 (0.0)	16	≤0.25	1	Kapur et al., 1994
	CAC-AAC	His-Asn	4	0 (0.0)	2	≤0.25	4	Ramaswamy et al., 2004
531	TCG-TTG	Ser-Leu	156	152 (97.4)	≥32	16	4	Donnabella et al., 1994
					≥32	8	4	
					≥32	4	42	
					≥32	2	56	
					≥32	1	46	
					16	0.5	1	
					8	≤0.25	1	
					4	≤0.25	1	
					2	≤0.25	1	
	TCG-CAG	Ser-Gln	1	0 (0.0)	8	≤0.25	1	Ramaswamy and Musser, 1998
533	CTG-CCG	Leu-Pro	3	0 (0.0)	≥32	0.5	1	Moghazeh et al., 1996
					2	≤0.25	2	
572	ATC-TTC	Ile-Phe	3	2 (66.7)	≥32	2	2	Yuen et al., 1999
					2	≤0.25		
**DOUBLE MUTATIONS**								
508	ACC-GCC	Thr-Ala	2	0 (0.0)	≥32	≤0.25	2	NA
516	GAC-GTC	Asp-Val						
509	AGC-ATC	Ser-Ile	2	0 (0.0)	8	≤0.25	2	NA
511	CTG-CCG	Leu-Pro						
511	CTG-CCG	Leu-Pro	5	0 (0.0)	≥32	≤0.25	3	Schön et al., 2013
515	ATG-GTG	Met-Val						
					2	≤0.25	2	
511	CTG-CCG	Leu-Pro	8	6 (75.0)	≥32	8	2	Laura et al., 2003
516	GAC-GGC	Asp-Gly						
					≥32	4	2	
					≥32	2	1	
					≥32	1	1	
					≥32	0.5	1	
					16	≤0.25	1	
511	CTG-CCG	Leu-Pro	2	0 (0.0)	8	≤0.25	1	Wang et al., 2011
526	CAC-CAA	His-Gln						
					2	≤0.25	1	
515	ATG-ATA	Met-Ile	1	0 (0.0)	16	0.5	1	NA
516	GAC-TAC	Asp-Tyr						
515	ATG-ATA	Met-Ile	1	1 (100.0)	≥32	2	1	Berrada et al., 2016
526	CAC-AAC	His-Asn						
516 522	GAC-GAG TCG-TTG	Asp-Glu Ser-Leu	2	0 (0.0)	≥32	0.5	1	Berrada et al., 2016
					4	≤0.25	1	
516	GAC-GCC	Asp-Ala	1	1 (100.0)	≥32	2	1	NA
526	CAC-AAC	His-Asn						
516	GAC-GGC	Asp-Gly	1	1 (100.0)	≥32	2	1	NA
532	GCG-GTG	Ala-Val						
526	CAC-CAA	His-Gln	1	0 (0.0)	16	≤0.25	1	NA
533	CTG-CCG	Leu-Pro						
WT	–	–	12	7 (58.3)	≥32	4	2	
					≥32	2	2	
					≥32	1	3	
					≥32	0.5	2	
					16	≤0.25	1	
					8	≤0.25	1	
					2	≤0.25	1	
Total	–	–	256	204 (79.7)	–	–	256	

a *The MICs of H37Rv aganisnt RIF and RFB were 0.2 and 0.05 mg/L, respectively*.

b *The reference represents the reference in which the mutation was first reported, while NA represents the mutation which is not found in published studies*.

### MICs and mutations of *rpoB* gene

Among the 256 RIF-resistant isolates analyzed, we identified 218 isolates (85.2%) with a single mutation, 26 (10.1%) with double mutations, and 12 (4.7%) without mutation in *rpoB* gene. The most frequently observed *rpoB* mutation was S531L (156/256, 60.9%), followed by H526D (11/256, 4.3%), H526R (9/256, 3.5%), H526C (7/256, 2.7%), H526L (6/256, 2.3%), and D516V (5/256, 2.0%). Of the 218 MTB isolates with a single mutation in *rpoB* gene, 188 (86.2%) were resistant to RFB. Resistance to both RIF and RFB was predominantly associated with S531L (152/188, 80.9%), H526D (11/188, 5.9%), H526R (9/188, 4.8%), H526C (7/188, 3.7%), and D516V (4/188, 2.1%), while some mutations within *rpoB* gene conferring RIF resistance were not responsible for RFB resistance such as L511P, D516G/Y, S522L, H526Y/L/D/N, S531Q, and L533P. In addition, only 9 out of 26 isolates (34.6%) with double mutations exhibited resistance to RFB, which was significantly lower than that of isolates with a single mutation (*P* < 0.01). Notably, although the single substitution of Leu511Pro and Asp516Gly did not result in RFB resistance, 75.0% (6/8) of the MTB isolates with these double mutations became resistant to RFB. A similar finding was observed in the RFB-resistant MTB isolates with double mutations of Asp516Gly and His526Asn. The RFB MICs of these isolates with double mutations ranged from ≤0.25 mg/L to 8 mg/L. In addition, we also found that 7 strains without nucleotide substitution in *rpoB* were resistant to RFB, accounting for 3.4% of RFB-resistant MTB isolates tested. Although all the single mutations have been reported by previous studies, we firstly identified 6 distinct double mutations conferring rifamycin resistance, as listed in Table 1.

### Distribution of MTB isolates with different *rpob* mutations

The proportions of MTB isolates harboring different mutant types were analyzed according to the drug susceptibility profile groups. As summarized in Table 2, compared with RR group (38.9%, 7/18), MDR-TB (63.5%, 106/167) had significantly higher proportion of isolates with mutations in codon 531 of *rpoB* gene (*P* = 0.04), while no significant difference was identified between MDR- and XDR-TB group (*P* = 0.83). In addition, there were no significant differences in the distribution of the isolates with other mutations among three groups (*P* > 0.05).

**Table 2:** Distribution of RIF-resistant isolates harboring different mutations within *rpoB* gene.

**Classification**	**No. of isolates (%)**				
	**531**	**526**	**Others**	**No**	**Total**
RIF-R	7 (38.9)	3 (16.7)	7 (38.9)	1 (5.6)	18 (100.0)
MDR-TB	106 (63.5)	31 (18.6)	23 (13.8)	7 (4.2)	167 (100.0)
XDR-TB	44 (62.0)	11 (15.5)	12 (16.9)	4 (5.6)	71 (100.0)
Total	157 (61.3)	45 (17.6)	42 (16.4)	12 (4.7)	256 (100.0)

We further compared the distribution and resistance levels to RIF of MTB isolates harboring mutations in different codon. Generally, the highest proportion of MTB isolates with high level of RIF resistance was identified in codon 531 (98.7%, 155/157), which was significantly higher than that of codon 511 (0.0%, 0/2, *P* < 0.001), 516 (62.5%, 5/8, *P* = 0.001), 526 (82.5%, 33/40, *P* < 0.001) and 533 (33.3%, 1/3, *P* = 0.001), respectively. On the contrary, there were no significant differences in the proportion of MTB isolates with high level of RIF resistance between other codon pairs (Table 3). Notably, only the strains with mutations in codon 531 (31.8%, 50/157) and 516 (37.5%, 3/8) correlated with high level of RFB resistance, and the statistically significant differences in the distribution of isolates classified into various resistant groups were observed in the strains with codon 511 (*P* = 0.002), 516 (*P* < 0.001), 526 (*P* < 0.001), and 533 (*P* < 0.001), when setting the strains with mutations in codon 531 as control. Similarly, the strains with mutations in codon 516 (37.5%, 3/4) had a higher proportion of high level of RFB resistance than those with mutations in codon 526 (0.0%, 0/40, *P* < 0.001; Table 4).

**Table 3:** Distribution of MTB isolates with different RIF-resistance level.

**Codon of mutation**	**No. of isolates with different RIF-resistance level^a^**		***P*** **value (codon versus codon)^b^**								
	**Low (%)**	**High (%)**	**511**	**513**	**514**	**516**	**522**	**526**	**531**	**533**	**572**
511	2 (100.0)	0 (0.0)	–	0.333	0.333	0.444	1.000	0.042	**0.000**	1.000	0.400
513	0 (0.0)	1 (100.0)	–	–	–	1.000	1.000	1.000	1.000	1.000	1.000
514	0 (0.0)	2 (100.0)	–	–	–	1.000	1.000	1.000	1.000	0.400	1.000
516	3 (37.5)	5 (62.5)	–	–	–	–	1.000	0.336	**0.001**	0.545	1.000
522	1 (50.0)	1 (50.0)	–	–	–	–	–	0.348	0.037	1.000	1.000
526	7 (17.5)	33 (82.5)	–	–	–	–	–	–	**0.000**	0.106	0.470
531	2 (1.3)	155 (98.7)	–	–	–	–	–	–	–	**0.001**	0.056
533	2 (66.7)	1 (33.3)	–	–	–	–	–	–	–	–	1.000
572	1 (33.3)	2 (66.7)	–	–	–	–	–	–	–	–	–

a *Low level resistance: MIC ≤ 4 mg/L; high level resistance: MIC > 4 mg/L*.

b *The highlighted P value represents that the difference between the two codons is significant [P value is less than the false discovery rate (FDR) 0.009]*.

**Table 4:** Distribution of MTB isolates with different RFB-resistance level.

**Codon of mutation**	**No. of isolates with different RFB-resistance level^a^**			***P*-value (codon versus codon)^b^**								
	**Susceptible (%)**	**Low (%)**	**High (%)**	**511**	**513**	**514**	**516**	**522**	**526**	**531**	**533**	**572**
511	2 (100.0)	0 (0.0)	0 (0.0)	–	0.333	1.000	0.600	1.000	0.106	**0.002**	–	0.400
513	0 (0.0)	1 (100.0)	0 (0.0)	–	–	1.000	0.600	1.000	1.000	1.000	0.250	1.000
514	1 (50.0)	0 (0.0)	1 (50.0)	–	–	–	1.000	1.000	0.015	0.026	0.400	0.600
516	4 (50.0)	1 (12.5)	3 (37.5)	–	–	–	–	0.667	**0.000**	**0.000**	0.618	0.364
522	1 (50.0)	1 (50.0)	0 (0.0)	–	–	–	–	–	0.528	0.074	0.400	1.000
526	12 (30.0)	28 (70.0)	0 (0.0)	–	–	–	–	–	–	**0.000**	0.037	1.000
531	5 (3.2)	102 (68.2)	50 (31.8)	–	–	–	–	–	–	–	**0.000**	0.138
533	3 (100.0)	0 (0.0)	0 (0.0)	–	–	–	–	–	–	–	–	0.400
572	1 (33.3)	2 (66.7)	0 (0.0)	–	–	–	–	–	–	–	–	–

a *Low level resistance: MIC ≤ 2 mg/L; high level resistance: MIC > 2 mg/L*.

b *The highlighted P value represents that the difference between the two codons is significant [P value is less than the false discovery rate (FDR) 0.014]*.

## Discussion

Although high-level cross-resistance between RIF and RFB has been repeatedly observed in MTB isolates, many studies have demonstrated that specific *rpoB* mutations are associated with differential resistance to RIF and RFB (Jamieson et al., 2014; ElMaraachli et al., 2015). Our data were in line with previous findings, which have consistently shown that the *rpoB* mutations S531L, H526D, H526R, H526C, and D516V conferred phenotypical resistance to both RIF and RFB, whereas amino acid substitution at codons L511P, D516G/Y, S522L, H526Y/L/D/N, S531Q, and L533P were associated with phenotypic resistance to RIF and susceptibility to RFB. In addition, we detected 11 different double mutations within *rpoB* from a total of 26 strains studied, including 3 infrequently encountered double mutations that have not been described elsewhere (Sandgren et al., 2009). Interestingly, a single specific mutation in codons 511, 516, and 526 was linked to the susceptibility to RFB for MTB, while the strains with these double mutations irrelevantly conferring RFB resistance produced RFB-resistant phenotype. The increased resistance to rifamycin contributes to the loss of binding affinity between rifamycin and RpoB protein (Pang et al., 2013a). The high MIC values of Ser531Leu and His526Asp mutants are due to the low affinities to the rifamycin molecules. For MTB isolates with specific mutations in codons 511, 516, and 526, we hypothesize that the structural alternations of the *rpoB* may be mediated by the presence of these amino acid substitutions. Despite being no correlated with RFB resistance, these structural alternations could result in loss of RFB susceptibility compared with isolates harboring wild-type *rpoB* genotype. Although the exact reason is unknown, the potential synergistic effect between double specific mutations may contribute to the conversion of RFB susceptibility among MTB isolates. It is important to noted that the majority of these RFB-resistant isolates with double mutations had low RFB MICs (≤4 mg/L), which may reflect the moderate synergistic effect of the two mutations within *rpoB*. Further structural analysis of RFB and these mutant RpoBs will be required to confirm our hypothesis.

Another interesting finding of our results was that the rate of RFB-resistant isolates with double mutations was significantly lower than that of isolates with a single mutation. One possible reason for this observation is that the double mutations identified in our study majorly consisted nucleotide substitutions located in codon 511, 515, 522, and 533 of *rpoB*, which confer low-level RIF resistance, while have no correlation with RFB resistance (Berrada et al., 2016). In view of the low-level RIF MICs of the strains with these single mutations, the high exposure to RIF may accelerate the occurrence of the second mutation. The acquisition of genetic mutation often comes with a cost to strain fitness (Mariam et al., 2004; Vogwill and MacLean, 2015). Briefly, the mutations resulting in higher level of drug resistance are associated with the higher loss of fitness in MTB isolates (Mariam et al., 2004). Hence, the MTB strains with single mutation conferring low-level resistance prefer to have a second accumulated mutation with less loss of fitness, which might be essential for survival from interspecific competitive pressures. This serves as a reasonable explanation for the high frequency of mutations conferring low-level resistance among double mutant MTB isolates. In addition, the proportion of strains with double mutations among RR group was significantly higher than that of MDR-TB group, indicating double mutations in the core region of *rpoB* gene lead to higher fitness cost than a single mutation, and a high rate of strains with double mutations are excluded during the period of the further accumulation of INH resistance.

Roll-out of molecular diagnostics provides an alternative for clinicians to obtain drug susceptibility results within shorter turn-around time than conventional DST (Pang et al., 2013b). Numerous commercial kits have been developed to analyze RIF susceptibility of MTB on the basis of detecting the core region of *rpoB* gene (Pang et al., 2013b, 2014). However, in light of the differential resistance to RIF and RFB with various *rpoB* mutations in MTB isolates, the WHO-recommended GeneXpert assay and GenoType MTBDR are not suitable for predicting RFB resistance due to failure to interpret the exact mutant types of *rpoB* gene. Specially, several previous studies have demonstrated that RFB-containing regimen produces more favorable efficacy against RFB-susceptible MDR-TB cases compared with RFB-resistant MDR-TB receiving other DST-guided regimen (McGregor et al., 1996; Jo et al., 2013). Therefore, accurate and rapid diagnostics designed for RFB is essential for guiding RFB-based therapeutic regimens and achieving favorable treatment outcome.

There were several obvious limitations in this study. First, the Clinical and Laboratory Standards Institute recommends the use of the standardized agar proportion method for susceptibility testing of MTB isolates. Although there were strong evidences that the excellent correlation was observed between the resistance determined by agar proportion method and microplate alamarBlue assay method (Chauca et al., 2007; Yu et al., 2011), the difference in methodology may interfere with the analysis of the relationship between *in vitro* phenotypic resistance and genotypic mutations. Second, the MTB isolates enrolled in this study were only collected from one clinical hospital in Beijing. As the National Clinical Center on TB, patients from different regions of China seek health care in our hospital. However, the bias in sample enrollment may result in loss of representativeness. Further experiments should be performed to explore the molecular characteristics among RFB-resistant isolates from different regions of China, especially the prevalence of strain with novel double mutation identified in our study. Third, due to the sample number of the strains with rare mutation patterns, they were excluded from statistical analysis. The relationship between these mutations and the level of drug resistance therefore is still unknown. There is increasing interest in the extent to answer this question by the recruitment of a large number of strains with rare mutations in the future.

In conclusion, our data demonstrate that various *rpoB* mutations are associated with differential resistance to RIF and RFB. A single specific mutation in codons 511, 516 and 522 was linked to the susceptibility to RFB for MTB, while the strains with these double mutations irrelevantly conferring RFB resistance produced RFB-resistant phenotype. In addition, compared with RR group, MDR-TB has significantly higher proportion of isolates with mutations in codon 531 of *rpoB* gene. Further structural analysis of RFB and the rpoBs with double mutations will extend our knowledge of the RFB resistance mechanism in MTB.

## Author contributions

WJ, YP, HH, and NC designed this study and wrote the manuscript. WJ, YP, ZZ, GJ, YM, and FH performed experiments. WJ, YP, JW, and RG interpreted the data. All authors approved the final version of the paper.

### Conflict of interest statement

The authors declare that the research was conducted in the absence of any commercial or financial relationships that could be construed as a potential conflict of interest.
